# Territory Tenure Increases with Repertoire Size in Brownish-Flanked Bush Warbler

**DOI:** 10.1371/journal.pone.0122789

**Published:** 2015-03-30

**Authors:** Canwei Xia, Chentao Wei, Yanyun Zhang

**Affiliations:** Ministry of Education Key Laboratory for Biodiversity and Ecological Engineering, College of Life Sciences, Beijing Normal University, Beijing, China; Utrecht University, NETHERLANDS

## Abstract

Song repertoire size is often cited as a classic example of a secondary sexual trait in birds. Models of sexual selection and empirical tests of their predictions have often related secondary sexual traits to longevity. However, the relationship between repertoire size and longevity is unclear. Using capture-mark-recapture studies in two populations of the brownish-flanked bush warbler *Cettia fortipes*, we found that males with a repertoire size of three maintained territory tenure for a longer duration than did males with a repertoire size of two. These results provide evidence that even a minimal difference in repertoire size can serve as a potential signal of territory tenure capability.

## Introduction

In about 70% of songbird species, males are able to produce multiple song types [1,2]. Song repertoire size is often cited as a classic example of a secondary sexual trait [3–5]. Most empirical studies have documented positive relationships between the degree of a secondary sexual trait and longevity [6]. However, it has also been argued that exaggerated expression of a sexual trait may detrimentally affect survival, as the traits are often costly [7,8]; thus, low-quality individuals with poor expression of the trait might exhibit higher survival [9]. In terms of bird song, the relationship between repertoire size and longevity is unclear. Many studies have found that older males have larger repertoires. However, the positive relationship may be caused by males adding new songs to their repertoires as they age, rather than males with larger repertoire sizes living longer [10,11]. Due to the difficulty in obtaining longevity data in free-living birds [12,13], direct tests of the relationship between repertoire size and longevity have only been conducted in a few species. In the great tit *Parus major*, males with larger repertoires survive longer [9,14,15]. In the song sparrow *Melospiza melodia*, repertoire size is significantly positively correlated with survival in populations in British Columbia [16,17] but not in populations from Washington [18] or Pennsylvania [19]. In the great reed warbler *Acrocephalus arundinaceus*, repertoire size is weakly positively related to lifespan in a German population but not in a Swedish population [20]. Given these conflicting results, additional fieldwork in this area is warranted.

**Fig 1:**
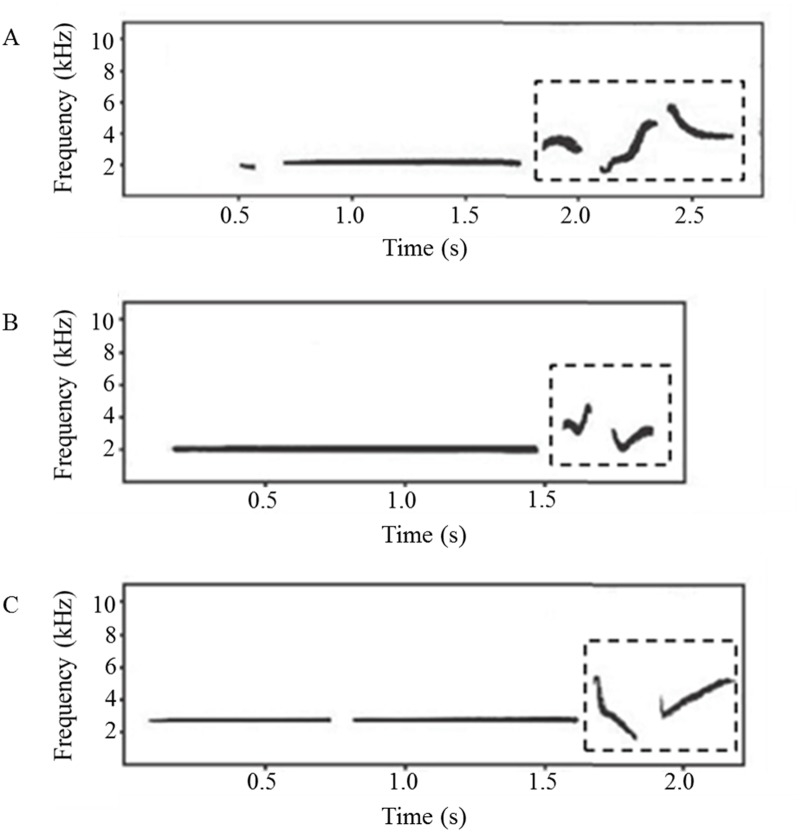
Spectrogram of songs from one male Brownish-flanked Bush Warbler. Dotted line frame indicating the terminal part. Number of notes within terminal part are different in (A) and (B), (A) and (C). Structures of notes within terminal part are different in (B) and (C).

In the present study, we investigated the relationship between repertoire size and longevity in two populations of the brownish-flanked bush warbler *Cettia fortipes*. This warbler is a small passerine that is widely distributed in Southeast Asia. Males sing clear, high-pitched songs from shrubs throughout the breeding season [21]. The songs consist of a whistle segment and a terminal segment (Fig 1). Different songs are categorized by the number and structure of notes in the terminal segment [22]. The repertoire sizes of males range from one to four, with two or three being the most common (>97% of individuals from the study populations), and they sing different songs in alternation or rotation (e.g., a-b-a-b; a-b-c-a-b-c; a-b-a-c-a-b-a-c) [22,23]. Repertoire size does not change during the breeding season in individual males [22]. Using territory tenure as a proxy for longevity (the validity of which will be addressed in the Discussion), we examined the relationship between repertoire size and longevity in the brown-flanked bush warbler. We found that even a minimal difference in repertoire size was related to increased longevity of this species.

## Materials and Methods

### Study Area and Target Species

We studied two populations of brownish-flanked bush warblers, one in the Dongzhai National Nature Reserve (DZ) in the Henan Province of southern China (31°28′–32°09′ N, 114°18′–14°30′ E) and one in the Kuankuoshui National Nature Reserve (KKS) in the Guizhou Province of southwest China (28°11′–28°15′ N, 107°08′–107°11′ E). These two populations are located approximately 900 km apart. The DZ consists of mature montane forests interspersed with small tea estates at 100–466 m elevation. The KKS consists of mature montane forests at 650–1762 m elevation. One tea farm that was abandoned in 2007 resides in the center of KKS at 1500–1600 m elevation. Additional information regarding DZ [24] and KKS [25] is described elsewhere.

The brownish-flanked bush warbler is a native species that inhabits scrubland dominated by tea plants (*Camellia sinensis*) in both DZ and KKS. Each territorial male defends a patch of tea plants. This warbler species has been monitored as part of a long-term study from 2007 to 2014 in DZ and from 2009 to 2011 in KKS. Nearly 200 and 140 territorial males inhabit our study areas within DZ [26] and KKS [22], respectively. In both populations, warblers begin nesting in late April and end around late August. Males guard their territory year-round in DZ, but they only sing from March to August. Males sing and guard their territory from April to August in KKS, but whether they continue to guard territory during the non-breeding season is unknown.

### Fieldwork

During the breeding season, we determined which habitats were most suitable to warblers in our study area. We then used playback of the species’ song to induce territory males to approach us during the daytime. Playback songs were recorded during the dawn chorus from three males in 2009 in KKS. Songs were played using a Teclast X18 Mp3 player (Teclast Co., China) connected to a Senway loudspeaker (Shenzhen Senway Amplifier Co., China). The amplitude of the playback song was 80 dB, measured using a CEL-240 sound level meter (Casella Co., UK) placed 1 m above the loudspeaker. The playback song rate was adjusted to one song per 10 s using the Goldwave 5.25 software (GoldWave Inc., Canada). This amplitude and song rate approximated those of natural songs. To minimize the familiarity effect, we avoided playing songs recorded from the target male or its immediate neighbors.

Males were trapped in mist nets as soon as they were observed guarding territory during playback, and each male was banded with a metal band and two plastic color bands. The territory position was marked using a MAP 60CSx GPS (Garmin Ltd., USA). We used a Tascam HD-P2 portable digital recorder (Tascam Co., Japan) and a Sennheiser MKH416 P48 external directional microphone (Sennheiser Co., Germany) to record songs during dawn chorus or after inducing singing with playback. Song type usage was similar during dawn chorus and inducing singing [23,27]. In total, 250 males were banded and recorded (S1 Table). Male territories as well as adjacent territories were revisited the following year. Over all study years, we marked 44 and 125 males in 2010 and 2011, respectively, in DZ, and 43 and 38 males in 2009 and 2010, respectively, in KKS. All males were banded during the first year that they occupied territory in our study areas.

### Song Analysis

We used Avisoft-SASLab Pro 4.3 (Avisoft Bioacoustics, Germany) to create spectrograms with fast Fourier transform lengths of 256 points, a hamming window with a frame size of 100% and an overlap of 50%, a frequency resolution of 86 Hz, and a time resolution of 5.8 ms. Repertoire size was determined by observing the spectrograms. According to Xia et al. (2010), different songs are categorized by differences in the number (Fig 1A and 1B, Fig 1A and 1C) or structure (Fig 1B and 1C) of notes in the terminal segment [22]. Based on repeated recordings (at intervals of about 4 days) of 20 banded males during the breeding season, we determined that the brownish-flanked bush warbler exhibits repertoire sizes ranging from one to four [22] and that they sing different songs in alternation or rotation [23]. Therefore, repertoire size could be determined from a recording containing as few as 10 continuous songs. In the present study, we analyzed at least 10 continuous songs for each male. To avoid the possibility of changes in repertoire size across different breeding seasons, we only used recordings from the year during which males were banded.

### Data Analysis

Territory tenure was divided into two categories: a single breeding season and more than one breeding season. Males that were seen only during the breeding season during which they were banded were defined as keeping a territory for a single breeding season, whereas males that were seen in the breeding season following that during which they were banded were defined as maintaining a territory for more than one breeding season.

Of the 250 males in the study, only four and two males exhibited repertoire sizes of one and four, respectively. Statistical analyses are difficult to apply to such small sample sizes; thus, males with repertoire sizes of one or four were omitted from the analysis, and only data for the remaining 244 males with repertoire sizes of two or three were analyzed. We also report results of analyses that pooled data for males with repertoire sizes of one and two and for males with repertoire sizes of three and four.

Analyses were performed using R v. 3.1.0 [28]. We used a generalized linear mixed model approach with the function glmer in the R package lme4 [29]. In the model, territory tenure (one or more than one) was the response variable with a binomial error distribution, repertoire size (two or three) was the fixed effect, and population (DZ or KKS) was the random effect. Significance was assumed at p < 0.05.

### Ethics Statement

Our research protocol was approved by the Animal Management Committee at the College of Life Sciences, Beijing Normal University under license number CLS-EAW-2013-010. The field studies did not involve endangered or protected species, and these studies were approved by the management bureaus of both the Dongzhai National Natural Reserve and the Kuankuoshui National Natural Reserve. Bird capture and banding were permitted by the National Bird-banding Center of China under license number H20110042.

## Results

**Fig 2:**
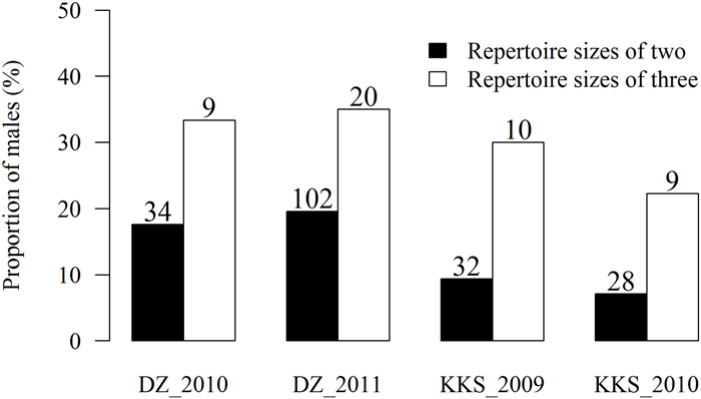
The proportion of males who kept their territory for more than one year. The proportion of males who kept their territory for more than one year in Dongzhai (DZ) and Kuankuoshui (KKS) with the total number of males shown above each bar.

Most males guarded their territory for only one breeding season (Table 1). In DZ, 20.9% and 22.1% of males banded in 2010 and 2011, respectively, were seen the following year. These rates were even lower in KKS, as only 14.3% and 10.8% of males banded in 2009 and 2010, respectively, were seen the following year. All males seen the following year maintained the same or an adjacent territory as when banded. Repertoire size significantly affected territory tenure (generalized linear mixed model, estimate = 0.920, SE = 0.373, 95% CI: 0.173–1.647, z = 2.46, p = 0.014). In both populations, males with a repertoire size of three exhibited longer territory tenures than did males with a repertoire size of two (Fig 2). If data were pooled for males with repertoire sizes of one and two and for males with repertoire sizes of three and four, the effect of repertoire size on territory tenure remained significant (generalized linear mixed model, estimate = 0.868, SE = 0.372, 95% CI: 0.124–1.592, z = 2.34, p = 0.020).

**Table 1:** The number of male Brown-flanked Bush Warblers monitored in Dongzhai (DZ) and Kuankuoshui (KKS).

Banded year	No. males	Territory tenure	
		One breeding season	More than one breeding season
**2010 in DZ**	43	34	9
**2011 in DZ**	122	95	27
**2009 in KKS**	42	36	6
**2010 in KKS**	37	33	4
**Total**	244	198	46

## Discussion

Based on 244 males from two populations of brownish-flanked bush warbler, we found that 22.2–35.0% of males with a repertoire size of three maintained their territory for more than one breeding season, whereas only 7.1–19.6% of males with a repertoire size of two exhibited territory tenures lasting longer than one breeding season (Fig 2). However, two possible biases must be considered before repertoire size can be concluded to affect male longevity in this species.

In the present study, we used territory tenure as a proxy for longevity. A territorial male that could not be found in his territory or elsewhere at the study site was presumed to have been unable to find a territory elsewhere. Therefore, dispersal may have biased our results. Based on our observational and banding data, dispersal by territorial males is rare in these two populations. In KKS, suitable habitat for brownish-flanked bush warbler is surrounded by at least a 2-km width of mature forest, which is unsuitable for this species due to the lack of shrubs. In DZ, the suitable habitat is somewhat continuous, and territorial males exhibit very low dispersal. In areas surrounding our research site, 477 brownish-flanked bush warblers were banded by the Dongzhai National Nature Reserve administration from 2010 to 2012. Only one bird that was re-banded in winter 2011 was banded during the 2011 breeding season in our study. Considering this low level of dispersal, the effect of dispersal on our results is likely to be minimal.

Second, we cannot exclude the possibility that a territorial male that could not be found in his territory was still alive and functioning as a floater. Because the primary habitat of the brownish-flanked bush warbler is dense shrubbery, in which it moves secretively, direct observations of this species are challenging [21]. Through playback of the species’ song, we were able to band and observe territorial males. However, floaters do not respond as intensely to playback as do territorial males; thus, they are only banded or observed by chance. A territory is a crucial resource for breeding [30]; therefore, if a territorial male loses the chance to breed due to the loss of a territory, we would classify this individual as dead, as its fitness is zero. In a study of the tree swallow *Tachycineta bicolor*, Kempenaers et al. (2001) found that 13% of 53 extra-pair young were fathered by floaters; thus, they hypothesized that floaters represent an alternate breeding strategy to territory holders [31]. In our study system, the question becomes whether a territorial male warbler that loses its territory could still achieve positive fitness through extra-pair fertilizations as a floater. While this issue is intriguing, we were unable to consider the possibility that a territorial male is still alive after losing territory due to the methodological constraints involved with monitoring floaters in our study populations of brownish-flanked bush warblers.

Here, we demonstrated a positive relationship between repertoire size and territory tenure in two populations of brownish-flanked bush warblers located 900 km apart. Previous studies have examined the relationship between repertoire size and survival in great tits [9,14,15], song sparrows [16–19], and great reed warblers [20]. Repertoire size ranges from 1 to 6 in the great tit [14], from 4 to 12 in song sparrows [16–18], and from 20 to 50 in the great reed warbler [20,32,33]. In our study populations, we compared the territory tenures of brownish-flanked bush warblers that only varied in repertoire size by one song (i.e., repertoire size was either two or three). Our results indicated that even this small difference in repertoire size could be an indicator of territory tenure in this species. Because of this small range in repertoire size, our findings provide key evidence of the role of repertoire size for territory tenure.

## Supporting Information

S1 Table Territory tenure and repertoire sizes of all banded and recorded males. (CSV)
